# Genomic subtyping of liver cancers with prognostic application

**DOI:** 10.1186/s12885-020-6546-8

**Published:** 2020-01-31

**Authors:** Zhenggang Wu, Xi Long, Shui Ying Tsang, Taobo Hu, Jian-Feng Yang, Wai Kin Mat, Hongyang Wang, Hong Xue

**Affiliations:** 1 HKUST Shenzhen Research Institute, 9 Yuexing First Road, Nanshan, Shenzhen, China; 2 Division of Life Science and Applied Genomics Center, Hong Kong University of Science and Technology, Clear Water Bay, Hong Kong, China; 3 Eastern Hepatobiliary Surgery Institute, Second Military Medical University, Shanghai, China; 4 Center for Cancer Genomics, School of Basic Medicine and Clinical Pharmacy, China Pharmaceutical University, Nanjing, China; 5 Jiangsu Key Lab of Cancer Biomarkers, Prevention and Treatment, Jiangsu Collaborative Innovation Center for Cancer Personalized Medicine, Nanjing Medical University, Nanjing, China; 6 Division of Life Science, Hong Kong University of Science and Technology, Clear Water Bay, Hong Kong, China

**Keywords:** AluScan, Genomic markers, Interstitial loss-of-heterozygosity, Mutation load, Mutational signature

## Abstract

**Background:**

Cancer subtyping has mainly relied on pathological and molecular means. Massively parallel sequencing-enabled subtyping requires genomic markers to be developed based on global features rather than individual mutations for effective implementation.

**Methods:**

In the present study, the whole genome sequences (WGS) of 110 liver cancers of Japanese patients published with different pathologies were analyzed with respect to their single nucleotide variations (SNVs) comprising both gain-of-heterozygosity (GOH) and loss-of-heterozygosity (LOH) mutations, the signatures of combined GOH and LOH mutations, along with recurrent copy number variations (CNVs).

**Results:**

The results, obtained based on the WGS sequences as well as the Exome subset within the WGSs that covered ~ 2.0% of the WGS and the AluScan-subset within the WGSs that were amplifiable by Alu element-consensus primers and covered ~ 2.1% of the WGS, indicated that the WGS samples could be employed with the mutational parameters of SNV load, LOH%, the Signature α%, and survival-associated recurrent CNVs (srCNVs) as genomic markers for subtyping to stratify liver cancer patients prognostically into the long and short survival subgroups. The usage of the AluScan-subset data, which could be implemented with sub-micrograms of DNA samples and vastly reduced sequencing analysis task, outperformed the usage of WGS data when LOH% was employed as stratifying criterion.

**Conclusions:**

Thus genomic subtyping performed with novel genomic markers identified in this study was effective in predicting patient-survival duration, with cohorts of hepatocellular carcinomas alone and those including intrahepatic cholangiocarcinomas. Such relatively heterogeneity-insensitive genomic subtyping merits further studies with a broader spectrum of cancers.

## Background

Primary liver cancer is the fifth most frequently diagnosed cancer, the second leading cause of cancer-related deaths in men and the sixth in women worldwide [1]. The major form of liver cancer is hepatocellular carcinoma (HCC), which accounts for ~ 75–90% of primary liver cancer cases, with intrahepatic cholangiocarcinoma (ICC) accounting for most of the remaining cases [2]. A relatively rare subtype of combined HCC and ICC (viz. HCC/ICC) that harbors both hepatocellular and biliary epithelial cancer pathologies is associated with poorer prognosis than either HCC or ICC. The main overall risk factor for liver cancers is virus infection: both hepatitis B virus (HBV) and hepatitis C virus (HCV) infections lead to chronic liver disease and possible subsequent cancer. HBV infection is associated with about half, and HCV infection with about 25%, of the HCC cases with considerable regional variations [3, 4]. There are also a range of non-viral risk factors for liver cancers, including alcohol intake, tobacco use and environmental exposures, which are consistent with variations in etiological and progression mechanisms.

Currently there are a number of staging systems and models with the goal of guiding the prognosis and treatment of HCC [5, 6]. Patient survival following diagnosis is mainly influenced by the three major interacting factors of tumor biology, patient’s underlying health and treatment program. Prognosis is commonly based on pathological presentations such as tumor size, number of tumor foci, vascular invasion, the presence or absence of metastasis, and the Child-Pugh scoring system. Liver cancer genomes have been investigated using WGS and whole exome sequencing (WES) [7, 8], and recurrent mutations have been found in such genes as *TP53*, *CTNNB1*, *PIK3CA* and *ARID1A* in HCC genomes [9, 10]. The GOH/CNV ratio among somatic mutations provides a parameter for classifying between different types of cancers [11]. Cancer-associated somatic copy number alterations (SCNAs) have been observed in cancers [12, 13] and applied to cancer prognosis [14, 15]. Recurrent germline CNVs identified by machine learning could also provide a basis to predict susceptibility to cancers including HCC [16].

Although SNV analyses of cancer genomes have long focused solely on GOH mutations, AluScan sequencing enabled the simultaneous amplification of myriads of inter-Alu sequences in the human genome through polymerase chain reaction (PCR) using Alu retrotransposon-consensus sequences as PCR primers, and revealed that a variety of tumors including HCCs and leukemia were massively burdened with interstitial copy-number neutral LOHs arising from a defective DNA-damage response [17, 18]. Examination of LOHs along with GOHs and CNVs also furnished support for a sequential model of cancer development [19]. In view of the importance of LOHs in cancer development, in the present study genomic parameters based on SNV, LOH, GOH and CNV contents, as well as SNV mutational signatures, of liver cancers have been analyzed regarding their utility for the prognosis of patient survival. For this purpose, the WGS sequence data on paired tumor-blood samples from liver cancer patients were analyzed, employing different mutational parameters as diagnostic criteria for stratifying the cancer samples into long and short patient-survival subgroups. The effectiveness of each criterion was assessed based on the statistical difference attained between the two subgroups in terms of their patient-survival periods. As well, because of the large costs in terms of expense and time required by WGS analysis, WGS sequences were compared to their Exome-subset and AluScan-subset sequences, in order to determine whether these much simpler subsets could be employed as for prognostic analysis in place of WGS.

## Methods

### Sequencing data and clinical information

The paired blood-tumor WGS data from the 110 Japanese liver cancers and their blood cell controls determined by Fujimoto et al [20] were downloaded from ICGC dataset version 18 Feb 2015 release (https://dcc.icgc.org), and allocated to three separate sets for analysis: the ‘110-Liver’ cohort comprising 85 HCC, 18 ICC and 7 HCC/ICC samples; the ‘85-HCC’ cohort comprising only the 85 HCC samples; and the ‘25-ICCG’ cohort comprising only the 18 ICC samples and the 7 HCC/ICC samples (Additional File 1: Table S1). White blood cell genomic DNA samples from the same patients were used as the controls in sequencing analyses for somatic variations in forms of SNVs and CNVs. This choice of blood over normal tissue as control was based on previous reports by us [19] and others [21, 22] that phenotypically normal tissue cells often contain many mutations, while blood cells even under cancerous situation, such as leukemia, bear minimal mutations [19]. Therefore, blood-tumor pairing could be a better design than normal-tumor pairing in somatic mutation analysis of genomic DNA, where tissue specific expression is not a major concern as in the case of RNA analyses. The SNV mutations, and their constituent GOH and LOH mutations, were called using the ‘UnifiedGenotyper’ module in GATK and filtered as described [17, 18]. Only the filtered segments that were present in both of the paired tumor and blood DNA samples were analyzed. Massive inter-pair variations were observed among the 110 liver blood-tumor pairs (ranging from 735 to 126,965 total SNVs, 445 to 21,139 GOHs, and 212 to 116,399 LOHs, Additional File 1: Tables S2).

Localized CNV calling was performed using 350-kb windows with the AluScanCNV algorithm [23] developed for improved CNV-calling from AluScan data and other types of MPS sequence data. Recurrent CNVs were defined by the cut-off frequency in the Poisson distribution of CNVs based on *p* < 0.05. Every recurrent CNV was used to define two patient groups, one in which the recurrent CNV was present and the other in which it was absent, and the Kaplan-Meier survival curves for the two groups were compared; any recurrent CNV that gave rise to statistically dissimilar survival curves in the log rank test (*p* < 0.01) was defined as a survival-associated recurrent CNV (srCNV).

To examine the potential utility of mutational signatures for cancer prognosis, the somatic SNVs in the WGS data of 110-Liver cohort were processed using the WTSI framework downloaded from http://www.mathworks.com/matlabcentral/fileexchange/38724 developed by Alexandrov et al. [24]; and 1000 iterations were performed setting the “total Signatures” parameter equal to 2 to generate two mutational signatures, α and β, from the input SNVs. In so doing, the major signature recognized by the WTSI framework was designated as Signature α. The SNVs not so designated were collectively designated as Signature β. Thus the percentage of SNVs assigned to the first signature yielded Signature α%, and the remaining SNVs assigned to the second signature yielded Signature β%. Signature α% represented the diagnostically useful signature parameter.

### Patient stratification for survival-duration prognosis

To stratify the tumor samples into the *long* and *short* patient-survival subgroups based on the genomic parameter of SNV burden (viz. total SNVs), LOH% or Signature α%, the tumor samples were arranged in a descending order according to the magnitude of the parameter, and the surv-cutpoint function in the ‘survminer’ R-package was performed under R environment to divide the tumors into two subgroups at different cut-points. The lengths of survival of the patients in the two subgroups were compared using the log-rank test, and the cut-point that yielded the lowest *p*-value between the two subgroups with respect to the lengths of patient survival was adopted as the optimal cut-point for dividing the two subgroups. To stratify the patients into the long survival and short survival subgroups based on srCNVs, the tumor samples were segregated into the low-srCNV and high-srCNV clusters using the pvclust R-package for hierarchical clustering with bootstrapping (*n* = 1000) [25]. The low-srCNV cluster corresponded to the long-suvival subgroup, and the high-srCNV cluster corresponded to the short-survival subgroup.

To correlate between patient survival and each of the 13 clinical parameters (top 13 rows in Additional File 1: Table S3), Kaplan-Meier analysis was conducted as described in the preceding section, in which the surv-cutpoint function was used to determine the optimal cut-point that yielded the minimum *p*-value in the log-rank test between the high-risk and low-risk subgroups. For Cox proportional hazards regression, the hazard ratio, viz. the exp. (coef) in Additional File 1: Table S3, of the age parameter was calculated in 1-year increments, and that of the SNV load parameter in increments of 1000. Each of the remaining parameters was normalized from 0 to 100%, and the hazard ratio was calculated in 1% increments (Additional File 1: Table S3). All correlation analyses were performed for one parameter at a time, i.e., single variant analyses.

### Experimental AluScan sequencing

Ten HBV-positive and five HBV-negative HCC samples and their respective blood cell controls were collected from Chinese Han patients with subject’s approval and institutional approval from the Eastern Hepatobiliary Surgery Hospital, Shanghai, China. Written informed consent was obtained from each patient who participated in this study. Subject recruitment and sample collection were approved by the institutional ethics review boards of National Center for Liver Cancer Research and the Eastern Hepatobiliary Surgery Hospital of Shanghai. Our research complied with the Declaration of Helsinki. Patient information including gender, age, virus status etc. are given in Additional File 1: Table S4. White blood cell DNA was prepared by phenol-chloroform extraction, and HCC tumor DNA was prepared using DNAzol Reagent (Life Technologies, USA). Experimental AluScan analysis was carried out as previously described [17–19]. In brief, multiplex inter-Alu PCR amplification was performed for each sample of 0.1 μg genomic DNA, using the four Alu consensus sequence-based primers AluY278T18 (5′-GAGCGAGACTCGTCTCA-3′), R12A/267 (5′-AGCGAGACTCCG-3′), AluY66H21 5′-TGGTCTCGATCTCCTGACCTC-3′) and L12A/8 (5′-TGAGCCACCGCG-3′), followed by sequencing library construction before subjected to next generation sequencing on the Illumina platform [17]. Illumina sequencing reads were mapped by BWA (Burrows-Wheeler Aligner, version 0.6.1) [26] to reference human genome hg19 downloaded from UCSC, followed by base recalibration and local realignment by GATK (Genome Analysis Tool-Kit, version Lite-2.1-8-gbb7f038) [27] according to the standard framework [28].

### The exome-subset and the AluScan-subset

The Exome-subset sequences within each WGS were identified based on the regions targeted in the Illumina TruSeq Exome kit, which covered ~ 2.01% of the human hg19 genome. The region information was listed in Additional File 3: Data S1. The AluScan-subset sequences within each WGS were identified based on the merged experimental AluScan sequences of fifteen HCC patients of Chinese origin from genomic regions that were covered by at least four reads with gaps less than 80 bp long in each of the fifteen samples, which covered ~ 2.14% of the human hg19 genome. The region information was listed in Additional File 4: Data S2. Three columns in Data S1 and S2 is the chromosome, the start site and the end site of the region respectively.

## Results

### Increased SNV load as stratifying criterion for survival

**Fig. 1:**
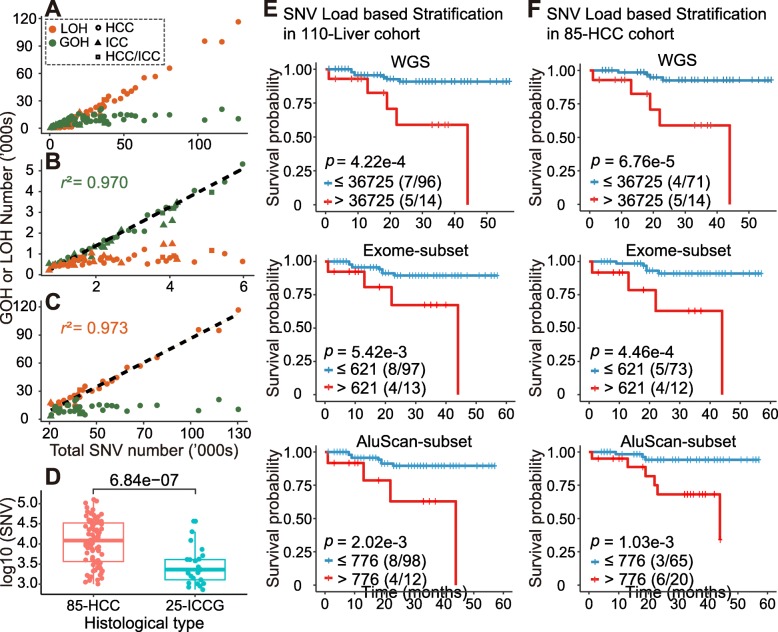
Relative numbers of GOH and LOH identified from mapped WGS data for 110 tumor-blood paired samples from Japanese liver cancer patients including 85 HCC, 18 ICC and 7 HCC/ICC, and survival analysis based on the SNV load. Numbers of GOH (green) and LOH (orange) were shown for (**a**) all 110 samples; (**b**) 49 samples with total SNV less than 6000; and (**c**) 35 samples with total SNV more than 20,000. Linear regression lines with respective coefficient of determination (*r*^*2*^) calculated from Pearson’s r statistic were shown for GOH in (B) and for LOH in (C). The relative numbers of GOH and LOH in the Exome-subset and AluScan-subset are given in Additional File 1: Table S2. (**d**) Comparison of the numbers of SNVs in the WGSs of 85-HCC and 25-ICCG (18 ICC + 7 HCC/ICC) using student’s t-test. Kaplan-Meier survival plots for two survival-probability subgroups in 110 liver cancer patients (**e**) and 85 HCC patients (**f**) stratified based on the numbers of SNVs in their WGSs (upper panels), Exome-subset (middle panels), and AluScan-subset (lower panels). Number of death out of the total number of patients in each group was shown in the parentheses. Censored patients were indicated by the tic marks on the survival curves. The optimal cut-point of SNV load employed to divide the patients into two subgroups was identified as the cut-point yielding the lowest *p*-value in the log-rank test (see *‘Patient stratification for survival analysis’* in Methods)

When the WGS data of the tumor and blood pairwise samples of the 110-Liver cohort were subjected to SNV analysis, SNV load and its constituent GOH and LOH mutation numbers varied substantially between samples (ranging from 735 to 126,965 SNVs, 445 to 21,139 GOHs and 212 to 116,399 LOHs, Additional File 1: Table S5), and there was no significant correlation between SNV load, i.e., the total number of SNVs in each tumor genome, with the clinical parameters of age at operation, viral status or tumor grade (Additional File 2: Figure S1). The average level of per genome SNV se of 110 liver cancers was 17,953 as detected by WGS. These loads of the 110 liver cancers fell into three categories, i.e., the low (below 6000; dominated by GOH as shown in Fig. 1b), the high (above 20,000; dominated by LOH as illustrated in Fig. 1c), and the medium (between 6000 and 20,000; not obviously dominated by either GOH or LOH) categories.

With the Exome-subset, the average per genome SNV load was 257 per genome, which was limited by the small genome regions occupied by exomes. However, with the AluScan subset, because only 2.14% of the whole genome was sampled in depth by AluScan, the 445 SNVs obtained had to be multiplied by 100/2.14, which equaled 20,794 per genome, on account of the extra density of SNVs in the AluScan-sampled regions. The results therefore showed that the AluScan-subset usefully captured genomic regions with higher mutation density than the genome average measured based on WGS (Fig. 1e and f).

There were more GOHs than LOHs in tumor genomes with low SNV loads, but more LOHs than GOHs in tumor genomes with high SNV loads (Fig. 1a). GOHs were dominant over LOHs when SNV load was equal to or below ~ 6000 (Fig. 1b), and LOHs were dominant over GOHs when SNV load exceeded ~ 20,000 (Fig. 1c). A large proportion of the LOHs were copy neutral (average = 69.5%), and over 90% of the LOHs were copy neutral in thirty of the tumor genomes (Additional File 2: Figure S2). For the 85 HCC cases, likewise GOHs were dominant in the low-SNV genomes, and LOHs were dominant in the high-SNV genomes (Additional File 2: Figure S3 A-C). The SNV loads in the 85 HCC tumor genomes were higher than those in the 25 ICCG tumor genomes (*p* < 10^− 6^, Fig. 1d).

When the SNV load of each tumor genome was employed as a criterion for stratifying the 110 liver cancer cases into a low-SNV subgroup (containing ≤36,725 SNVs per sample) and a high-SNV subgroup (containing > 36,725 SNVs per sample), survival analysis by means of the log rank test indicated that the low-SNV subgroup was associated with longer patient survival compared to the high-SNV subgroup with respect to liver cancer-specific deaths (12 deaths among 110 patients) with *p* = 4.22e-4 (i.e. 4.22 × 10^− 4^) between the two subgroups (Fig. 1e top panel). This was similarly the case with respect to the liver cancer-specific deaths in the 85-HCC cohort (9 deaths among 85 patients) with *p* = 6.76e-5 (Fig. 1f top panel). When total deaths instead of liver cancer-specific deaths were considered, the *p*-values were somewhat higher, i.e. 4.50e-3 for the 110-Liver cohort with 15 deaths among 110 patients, and 2.12e-4 for the 85-HCC cohort with 10 deaths among 85 patients (Additional File 2: Figure S4). When the Exome-subset and AluScan-subset sequences were stratified into long and short survival subgroups using SNV load as the stratifying criterion, the results shown in Fig. 1e and f. Compared to WGS, AluScan and Exome subsets each yielded higher, or less significant, *p*-values between the two subgroups stratified based on SNV load (for 110-Liver cohort, *p* = 4.22e-4 with WGS, 5.42e-3 with Exome-subset, 2.02e-3 with AluScan-subset). The two subsets (Data S1 and S2) each involved about 50 times less sequencing data than WGS, nonetheless still giving rise to significant results in survival prognosis, with SNV load as the stratifying parameter. That the optimal cut-point for stratifying the patients into two subgroups of significantly different survival durations was identical for both the 110-Liver cohort (Fig. 1e) and the 85-HCC cohort (Fig. 1f) suggests that the method of SNV load-based stratification for survival duration prognosis was robust and resistant to sample heterogeneity.

### Association of high LOH% with poor prognosis

**Fig. 2:**
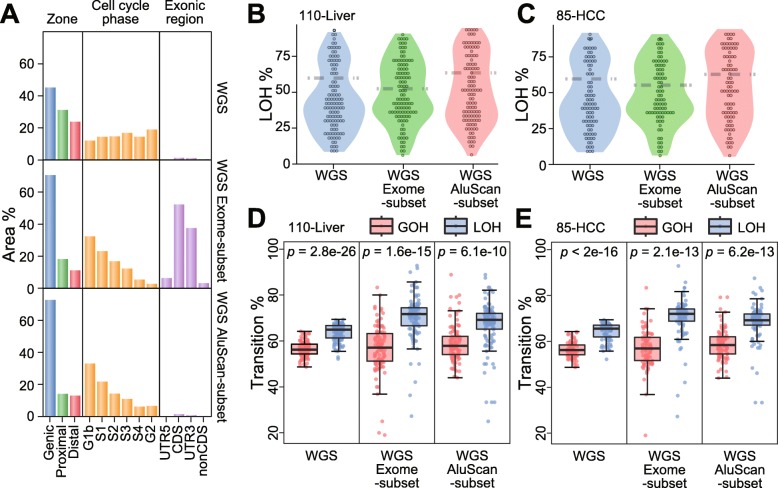
Comparison of the properties of WGS sequences of the 110-Liver cohort with the Exome-subset and the AluScan-subset. (**a**) Distribution of WGS, Exome-subset and AluScan sequences (in % of sequence regions) among the Genic, Proximal and Distal zones, with *p* values denoting the difference between GOHs and LOHs. among different cell cycle phases; and among different exonic regions. (**b**, **c**) Violin plots of LOH% (percentages of somatic LOHs relative to SNV load) in the 110-Liver and 85- HCC cohorts for three types of sequence data. (**d**, **e**) The percentages of transitional somatic GOHs and LOHs relative to total somatic GOHs and LOHs (red and blue boxes) for three types of sequence data, with the significant difference between GOHs and LOHs from paired t-tests expressed by the *p*-values for the 110-Liver and 85-HCC cohorts

To determine whether SNV-based prognosis could be usefully performed with a sequence subset instead of the entire WGS sequence, all the Exome sequences represented in the Illumina TruSeq Exome kit and covering ~ 2.0% of the genome (data S1) were extracted from the WGS and analyzed as the Exome-subset sequences. Similarly, the experimental AluScan sequences obtained from the 15 Chinese HCC patients as described in Materials and Methods (data S2) defined the AluScan amplifiable sequences in the WGS that covered ~ 2.1% of the genome, and extraction of these sequences from each WGS of the 110 Liver tumor samples yielded the AluScan-subset sequences. In this regard, it may be noted that DNA sequences in the human genome are classified into Genic zones enriched with gene sequences, Proximal zones adjacent to genes and enriched with enhancers, and Distal zones relatively depleted in genes [29]; into different cell-cycle phases regarding the timing of DNA duplication; and into exonic regions containing coding sequences (CDS), their adjoining untranslated regions (UTRs), and nonCDS segments on the RNA transcripts. Since the WGS sequence, the Exome-subset sequences and the AluScan-subset sequences differ from one another in terms of (i) the proportions of Genic zones, Proximal zones and Distal zones they contain; (ii) the profile of duplication times of their DNAs in the cell cycle; and (iii) the abundance of exonic regions found in them (Fig. 2a), it follows that the WGS, Exome-subset and AluScan-subset sequences within any tumor would contain nonidentical SNV profiles, as illustrated by their dissimilar percentages of transitional SNVs (Fig. 2b and c) and dissimilar SNV violin-plots of LOH% (Fig. 2d and e). Interestingly, the six violin plots all exhibited upper and lower bulges, which would be consistent with the presence of at least two different underlying mechanisms for SNV production.

**Fig. 3:**
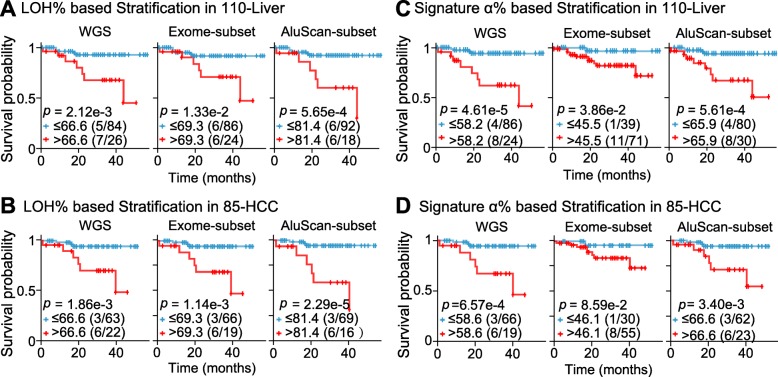
Kaplan-Meier survival plots of two survival-probability subgroups of the 110-Liver and 85-HCC cohorts estimated from WGS, Exome-subset or AluScan-subset sequences, each stratified based on (**a**) LOH% in 110-Liver cohort; (**b**) LOH% in 85-HCC cohort; (**c**) Signature α% in 110-Liver cohort; and (**d**) Signature α% in 85-HCC cohort. Low LOH% or Signature α% curve represented in blue, and high LOH% or Signature α% curve in red. Sample sizes of the two stratified groups were indicated in parentheses, and censored patients indicated by tic marks on the curves

Since high SNV loads were associated with increased death rate in the 110-Liver or 85-HCC cohorts (Fig. 1e and f), and with high fractions of LOH among the SNVs (Fig. 1a-c), the LOH% in the observed SNVs might be expected to furnish an alternative criterion for stratifying tumor samples in terms of patient survival times. When LOH% was employed as a stratifying criterion to divide patient samples in the 110-Liver cohort into high-LOH% and low-LOH% subgroups using the WGS, Exome-subset or AluScan-subset sequences based on liver cancer-specific deaths, the log rank test indicated that the low-LOH% subgroup was in each instance longer surviving than the high-LOH% subgroup, with *p* = 2.12e-3, 1.33e-2 or 5.65e-4 based on WGS, Exome-subset or AluScan-subset sequences respectively for the 110-Liver cohort (Fig. 3a); and *p* = 1.86e-3, 1.14e-3 or 2.29e-5 based on WGS, Exome-subset or AluScan-subset sequences respectively for the 85-HCC cohort (Fig. 3b). Therefore, the AluScan-subset sequences outperformed prognostically the WGS and Exome-subset sequences.

### Prognostic application of mutational signatures

**Fig. 4:**
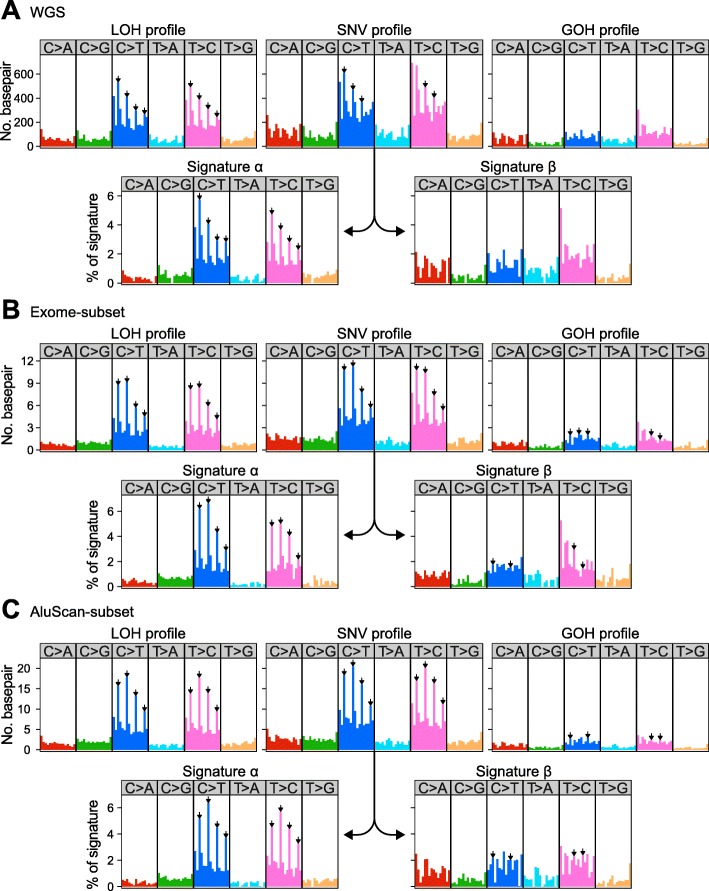
Mutational profiles of total SNVs, GOH or LOH in 110-Liver cohorts, along with Signatures α and β resolved from the total SNV profile. (**a**) WGS SNV mutations; (**b**) Exome-subset mutations; and (**c**) AluScan-subset mutations. All profiles were displayed using the 96-trinucleotide contexts where the mutated base was flanked by the 3′ and 5′ neighboring bases. The frequency bars represent the numbers of six types of substitution mutations, viz. C > A, C > G, C > T, T > A, T > C and T > G. Inverted arrows above the bars were typical of Signature α where mutations at NCG trinucleotides constituted the tallest peaks in the C > T or T > C boxes

There are multiple chemical pathways for SNV production in cells, and different pathways have been correlated with distinctive SNV mutational signatures, with decreased accuracy of deciphering the signatures for an increased number of resolved signatures [24, 30]. In the mutation profiles displayed by the 110-Liver cohort (Fig. 4a), mutations of C to T in the C > T box, and mutations of T to C in the T > C box, were dominant in the LOH and SNV profiles but not in the GOH profile. Upon resolution of the SNV profile into Signatures α and β, the Signature α resembled the LOH profile: both of them were marked by the signature comprising four inverted arrows pointing to the enhanced mutations at the NCG triplets in the C > T box, and NTG triplets in the T > C box. The SNV profiles of the Exome-subset and AluScan-subset could be resolved similarly (Fig. 4b and c). To determine whether these mutational signatures might be useful for prognostic purpose, the SNVs in the 110-Liver and 85-HCC cohorts were each resolved into Signatures α and β. Signature α resembled the LOH profile, no matter the SNVs were obtained from WGS (Fig. 4a), Exome-subset (Fig. 4b), or AluScan-subset (Fig. 4c). However, the less characteristic Signature β was apparently a mixture of GOHs and LOHs. The estimated Signature α% for each sample was employed as a stratifying criterion to divide patient samples in the 110-Liver or 85-HCC cohorts into high-α% and low-α% subgroups using the WGS, Exome-subset or AluScan-subset sequencing data. Based on liver cancer-specific deaths, the log rank test indicated that the low-α% subgroups were longer surviving than the high-α% ones for both the 110-Liver and 85-HCC cohorts, with *p* = 4.61e-5, 3.86e-2 or 5.61e-4 for the WGS, Exome-subset or AluScan-subset sequences of the 110 liver cancer samples respectively (Fig. 3c); and *p* = 6.57e-4, 8.59e-2 or 3.40e-3 based on the WGS, Exome-subset or AluScan-subset sequences of the 85-HCC cohort respectively (Fig. 3d). Therefore, the WGS sequences were the most useful prognostically followed by the AluScan-subset and the Exome-subset for both the 110-Liver cohort and the 85-HCC cohort.

### Nature of somatic mutations in AluScan-subset sequences

**Fig. 5:**
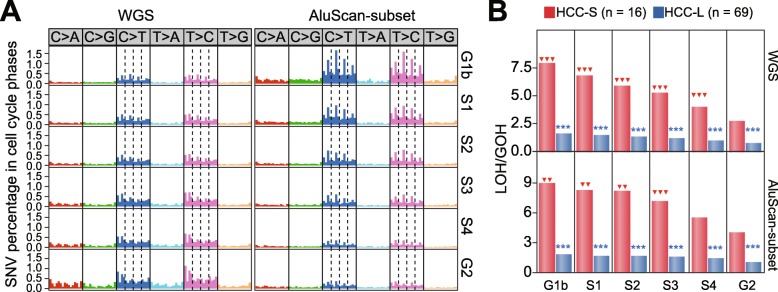
(**a**) Comparison of the SNV profiles in the 110-Liver cohort that belonged to the six different cell cycle phases. (**b**) Comparison of the LOH/GOH ratios observed in the short-survival (HCC-S) or long-survival (HCC-L) subgroups within DNA regions duplicated in the different cell cycle phases. The red triangles indicate significant difference of the LOH/GOH ratio in the G2 phase relative to the earlier five phases. The blue asterisks indicate significant difference of the LOH/GOH ratio between the HCC-S and HCC-L groups (two arrows or asterisks, *p* < 0.01; three arrows or asterisks, *p* < 0.001)

Based on the *p*-values distinguishing between the stratified subgroups based on SNV load, LOH% or Signature α%, the Exome-subset yielded generally higher *p*-values than WGS and AluScan-subset sequences, and were thus the least useful. However, although WGS outperformed AluScan-subset in the stratifications based on SNV load or Signature α% (Fig. 1e, f, 3c and d), the AluScan-subset covering only ~ 2.1% of the genome outperformed WGS in the stratifications based on LOH% for both the 110-Liver and 85-HCC cohorts (Fig. 3a and b). The reason for this unexpected prognostic utility of LOH% in the AluScan-subset is that the AluScan sequences were enriched in genic regions and regulatory elements [17, 18], resulting in a greater concentration of cancer SNVs in the AluScan-subset DNA compared to WGS DNA regardless of the duplication-phase (G1b to G2) of the DNA (Fig. 5a). There were also more LOHs relative to GOHs in the short-survival 85-HCC (HCC-S) cases compared to the long-survival (HCC-L) cases (Fig. 5b).

### Usage of recurrent somatic CNVs for survival prognosis

**Fig. 6:**
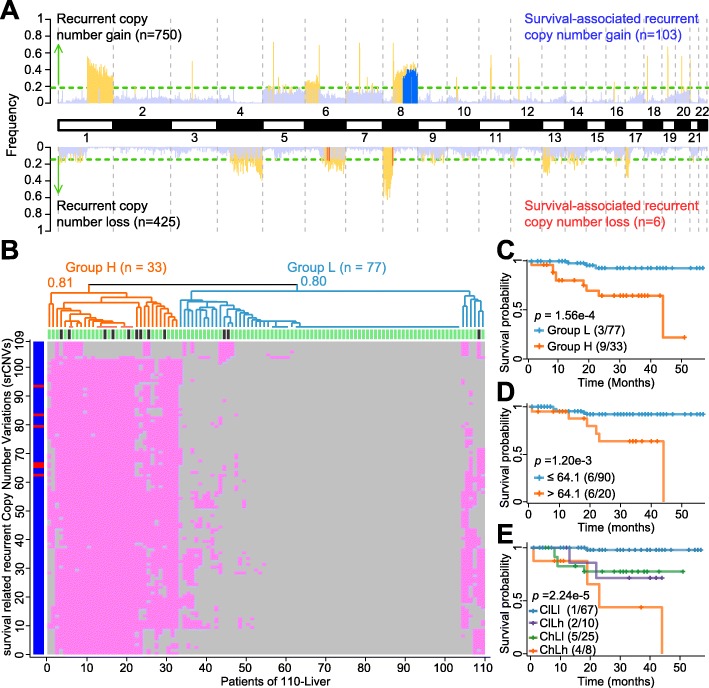
Analysis of srCNVs in the 110 Liver samples. (**a**) Chromosomal distribution of recurrent CNVs and srCNVs. Recurrent CN-gains are represented by yellow upward bars, recurrent CN-losses by yellow downward bars, srCN-gains by upward blue bars, and srCN-losses by downward red bars. The green horizontal lines mark the frequency thresholds for significant recurrence (0.19 for CN-gain and 0.15 for CN-loss; *p* < 0.05), and CN-gains and losses that fell below these thresholds were represented by light blue columns. All srCNVs were located on chromosomes 6 and 8. (**b**) The srCNVs were employed for hierarchical clustering of the 110 liver cancer samples. The approximately unbiased *p*-values after 1000 times bootstrapping for the high-srCNV group (Group H) and low-srCNV group (Group L) were given at the respective nodes. (**c**) Kaplan-Meier survival plots for Groups H (orange) and Group L (blue). (**d)** Kaplan-Meier survival plots stratified based on LOH% comprising only copy- neutral LOHs. (E) Kaplan-Meier survival plots based on LOH% and srCNV. LOH% and srCNV were jointly applied to the 110-Liver cohort to stratified them into the four subgroups according to Kaplan-Meier survival plots. Censored patients were indicated by tic marks on the survival curves

For the 110-Liver cohort, 1175 recurrent somatic CNVs were identified from the WGS data. Of these, 109 were significantly associated with survival (*p* < 0.01), and thereby designated as survival-related ‘srCNVs’: five were srCN-losses in the long arm of chromosome 6 at 6q16, one an srCN-loss in the short arm of chromosome 8 at 8p11.21, and 103 were srCN-gains located in the long arm of chromosome 8 from 8q21.3 to 8q24.3 (Fig. 6a). To divide the tumor samples into two subgroups with unequal survival probabilities, the patient-survival status for each of the 110 samples are plotted in Fig. 6b along the x-axis at the top of the square, with a short vertical bar showing the survival status of each patient at the 50-month time point as alive (green) or deceased (black). The different srCNVs are plotted along the y-axis, with the six srCN-losses in the form of red horizontal bars, and the 103 srCN-gains in the form of vertically-merged blue horizontal bars. The presence of any particular srCNV in a sample is represented by a small pink square, and the absence by a small grey square. Hierarchical clustering was employed to stratify the tumor samples into a cluster high in srCNV content (Group H, *n* = 33) from a cluster low in srCNV content (Group L, *n* = 77), as indicated on top of the diagram. When the Kaplan-Meier survival curves for Groups H and L (Fig. 6c) were analyzed, patient survival in Group L was significantly longer compared to Group H (*p* = 1.56e-4), thereby establishing the srCNV parameter as a useful stratifying criterion for survival prognosis.

Since either srCNV (Fig. 6c) or LOH% (Fig. 3a) could be employed as a stratifying criterion for prognostic purpose, the question arose whether the effectiveness of LOH% as stratifying criterion might be dependent entirely on the elimination of heterozygous residues in the genome by CN-losses. To examine this possibility, all the CN-gains and CN-losses in the WGSs of 110-Liver cohort were deleted prior to stratifying the 110-Liver cohort based on the remaining CN-neutral LOH%. The results obtained (Fig. 6d) enabled nonetheless a significant distinction between a long-survival (upper curve) and a short-survival (lower curve) subgroups with *p* = 1.20e-3, demonstrating that the LOH% and srCNV stratifying criteria were based on overlapping but non-identical genomic elements. As well, when LOH% and srCNV were jointly applied to the 110-Liver cohort to divide them into the low CNV-low LOH (ClLl), low CNV-high LOH (ClLh), high CNV-low LOH (ChLl) and high CNV-high LOH (ChLh) subgroups, the four subgroups were distinguishable from one another with an overall *p* = 2.24e-5 (Fig. 6e).

When srCNV analysis was performed on the 85-HCC cohort, 70 srCNVs were obtained including one srCN-loss in the long arm of chromosome 6 at 6q16, seven srCV-loss in the short arm of chromosome 8 at 8p11.21, and 62 srCN-gains in the long arm of chromosome 8 from 8q21.3 to 8q24.3 (Additional File 2: Figure S5). Hierarchical clustering of the 85 samples into a high-srCNV group (Group H) and a low srCNV group (Group L) gave rise to different prognosis curves for the two groups with *p* = 1.03e-3 (Additional File 2: Figure S5B).

Regarding the stratification of patient samples into the long and short-survival subgroups employing SNV load, LOH%, α% or srCNV as stratifying criterion, the question also arose with respect to the extent such stratification could be influenced by a biased enrichment of metastasis in the short-survival subgroup. Accordingly, Fisher’s exact test was employed to assess the possible correlation between total SNV load, LOH% or Signature α% on the one hand, and the presence of hepatic vein and/or portal vein metastasis on the other in the 85-HCC cohort, which included a higher percentage of metastasis than the 110-Liver cohort. The results indicated only marginal positive correlation between them (Additional File 1: Table S5). However, the 25-ICCG cohort was heavily enriched with portal vein metastasis (14 out of 25 cases), hepatic vein metastasis (10 out of 25) or both (8 out of 25). When the associations of various clinical and mutational parameters with the length of patient survival were analyzed using Kaplan-Meier log rank test and Cox regression, significant associations were found with moderate *p*-values for the clinical parameters of gender, portal vein invasion, hepatic vein invasion and tumor size, and with lower *p*-values for the mutational parameters of SNV load, LOH% and Signature α% (Additional File 1: Table S3).

### Experimental AluScan-captured sequences

**Fig. 7:**
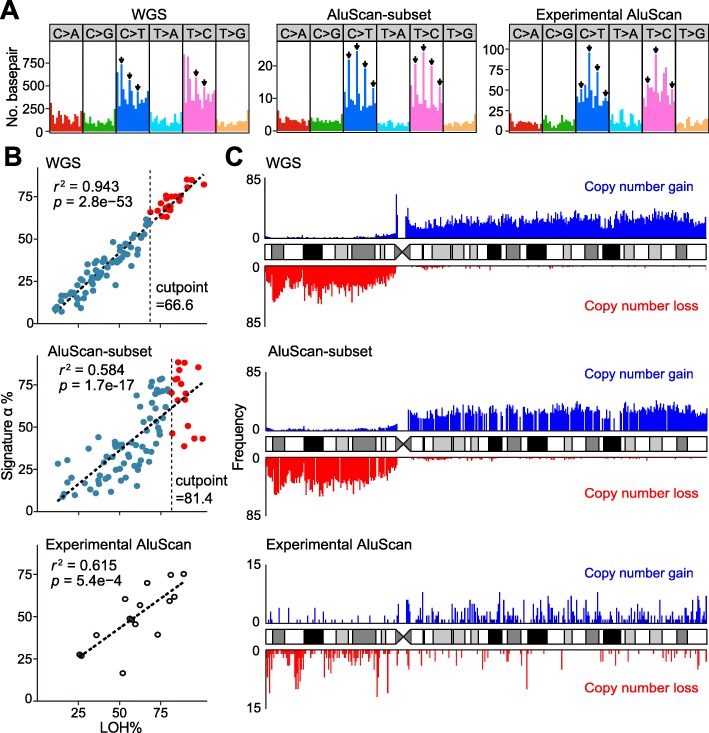
AluScan analysis of HCC patients. Comparison of (**a**) SNV profiles in WGS, AluScan-subset and Experimental AluScan sequences. (**b**) Correlations between Signature α%. (**c**) CN-gains and losses in WGS, AluScan-subset and experimental AluScan sequences on chromosome 8. The CNV frequencies in 350-kb windows were shown as bars above and below the chromosome ideogram for CN-gains (blue) and CN-losses (red), respectively.

When experimental AluScan sequencing was performed on fifteen Chinese HCC patients, analysis revealed 1106 somatic SNVs in the AluScan sequences that were amplified from all the paired blood-tumor paired samples (average capture of 13.8 Mb at read depth ≥ 8, Additional File 1: Table S6) without any significant correlation between somatic SNV density and the clinical parameters of age at operation, viral status, or tumor grade. When these experimental AluScan-sequence pairs were compared with both the blood-tumor WGS pairs from the 110-Liver cohort, and the blood-tumor AluScan-subset pairs extracted from the WGS pairs, the three sets of sequences displayed similar SNV profiles inclusive of both GOHs and LOHs. The main dissimilarity between these profiles was that the SNVs in the C > T and T > C boxes of the WGS profile were marked by altogether five inverted arrows, those in the AluScan-subset profile were marked by eight inverted arrows, and those in the experimental AluScan profile were marked by seven inverted arrows (Fig. 7a). The Signature α% was linearly correlated with LOH% in all three cases (Fig. 7b), and chromosome 8q in all three cases showed an abundance of CN-gains whereas chromosome 8p showed an abundance of CN-losses (Fig. 7c). That the peaks of CN-losses on chromosome 8q were distinctly shorter in the WGS and AluScan-subset from the Japanese liver cancer samples compared to the experimental AluScans from the Chinese liver cancer samples could be attributed at least in part to ethnic genomic differences.

## Discussion

Prognostic models are important to the treatment of cancers by providing information that facilitates the selection and monitoring of treatment modalities. Gene-specific markers such as estrogen/progesterone receptors (ER/PR) and human epidermal growth factor receptor 2 (HER2) in breast cancer [31], carcinoembryonic antigen (CEA) in colorectal cancer [32], *MYCN* in neuroblastoma [33], *KRAS* in pancreatic ductal carcinoma [34], *BRAF* in melanoma [35], and *EGFR* in lung adenocarcimona [36] have found valuable prognostic applications. The present study showed that the generalized, non-gene specific mutational parameters SNV load, LOH%, Signature α% and srCNV content (and expectedly their closely related parameters such as GOH%, LOH/GOH ratio, Signature β%, srCN-gains and srCN-losses) provide stratifying criteria for separating tumors into the long patient survival and short patient survival subgroups. Since the recurrent CNVs useful for predicting a subject’s propensity to cancer vary with the ethnic group [16], it would be necessary, in employing SNV load, LOH%, Signature α% or srCNV content to stratify prognostically a test patient’s tumor sample, to compare the test sample to standard stratified subgroups of the same type of cancer and from the same ethnic group as the test patient until indicated otherwise by available data.

In stratifying the 110-Liver and 85-HCC cohorts employing SNV load, LOH%, Signature α% or srCNV content as stratifying criterion, the results obtained from WGS data, AluScan-subset data and Exome-subset data indicated that the Exome-subset largely did not provide statistical distinction with sufficiently low *p*-values between the long-survival and short-survival subgroups, possibly on account of the relative paucity of cancer SNVs in the exomic regions. In the case of WGS and the AluScan-subset, low *p*-value were obtained based on WGS for all four stratifying criteria tested. On the other hand, the AluScan-subset surpassed the WGS data only when LOH% served as the stratifying criterion. Accordingly, where the performance of WGS sequence determination on a pair of blood-tumor paired samples per prognosis is unaffordable in terms of the time and labor costs needed, use of experimental AluScan data and LOH% as stratifying criterion would enable cost reduction compared to WGS, with the advantage that the method requires only submicrograms of DNA sample per analysis compared to the larger DNA sample size needed for WGS.

Previously, it was found that the nonsynonymous GOH type of SNV mutations were correlated with sensitivity to PD-1 blockade in cancer immunotherapy, with an association between increased GOH load and improved objective response, durable clinical benefit and progression-free survival [37–39]. While these findings might appear to depart from the present findings that increased SNV load or LOH% was correlated with decreased survival in the 110-Liver and 85-HCC cohorts (Fig. 1a-1b and 3a-3b), the difference was only an apparent one insofar that the effectiveness of PD-1 blockade depends on the failure of the cancer cell under onslaught by a specific therapeutic protein, whereas the shortened length of patient survival is the outcome of the prevalence of cancer cell over the host.

## Conclusions

In conclusion, because different types of cancers are caused by dissimilar oncogenic factors and mutational pathways, it was surprising that the generalized genomic variables of SNV load, LOH% and Signature α%, and srCNV could be significant correlates of the probability of survival against clinical cancers. A possible explanation might be that, while a cancer may be initiated by a small number of somatic mutations, its progression to outright malignancy often requires the continual accumulation of a large number of SNV, LOH and CNV mutations [18, 19], which is in accord with the large number of cancer-related genes discovered. Moreover, extensive double-strand DNA break repair by gene conversion may result in global genomic changes [18], and impact the genomic parameters as measured in this study. Consequently, these generalized genomic parameters represent significant determinants of the course of cancer, provide important stratifying criteria for prognosis, and may be generally useful as genomic markers in cancer subtyping. Further analysis of different types of cancers will indicate whether the prognostic utility of these genomic parameters may be extended to cancers besides hepatocellular carcinomas, and how their prognostic accuracy may vary with the stage of cancer when the prognosis is made.

## Supplementary information

**Additional file 1. Supplementary Tables S1-S6. Table S1.** Clinical and pathological information on 110-Liver cohort. **Table S2.** SNV numbers in different sequence datasets on 110-Liver cohort. **Table S3.** Survival analysis of clinical and genomic factors for patients in different cohorts of liver cancers. **Table S4. **Clinical and pathological information on 15 Chinese HCC patients. **Table S5.** Association between characteristics of somatic variations and clinical features in different cohorts of liver cancers. **Table S6.** Experimental AluScan analysis of 15 Chinese HCC patients.

**Additional file 2. Supplementary Figures S1-S5. Figure S1.** Correlation of SNV load with age, viral status and tumor grade in the 110-Liver cohort. **Figure S2.** Numbers of patients with different percentages of copy neutral LOHs in the 110-Liver cohort. **Figure S3.** Numbers of GOHs and LOHs identified from mapped WGS data for the 85-HCC cohort. **Figure S4.** Kaplan-Meier survival plots based on mutation loads measured by numbers of SNVs, GOHs or LOHs. **Figure S5.** Analysis based on the recurrent CNVs in 350-kb windows in the 85-HCC cohort.

**Additional file 3.** **Supplementary Data S1.** Exome-subset regions in BED format.

**Additional file 4.** **Supplementary Data S2.** AluScan-subset regions in BED format.

## Data Availability

All data generated or analyzed during this study are included in this published article and its additional files listed below:

## Abbreviations

CNV: Copy number variation
GOH: Single nucleotide variation with gain-of-heterozygosity
HBV: Hepatitis B virus
HCC: Hepatocellular carcinoma
HCV: Hepatitis C virus
ICC: Intrahepatic cholangiocarcinoma
ICGC: International cancer genome consortium
LOH: Single nucleotide variation with loss-of-heterozygosity
SNV: Single nucleotide variation
srCNV: Survival-associated recurrent copy number variation
WES: Whole exome sequencing
WGS: Whole genome sequencing
