# Supplementary material for: Genomic subtyping of liver cancers with prognostic application
Source: BMC Cancer. 2020 Jan 31;20:84. doi: 10.1186/s12885-020-6546-8 (PMC6995214; doi:10.1186/s12885-020-6546-8)
Supplement: Supplementary file 2 — Additional file 2. Supplementary Figures S1-S5. Figure S1. Correlation of SNV load with age, viral status and tumor grade in the 110-Liver cohort. Figure S2. Numbers of patients with different percentages of copy neutral LOHs in the 110-Liver cohort. Figure S3. Numbers of GOHs and LOHs identified from mapped WGS data for the 85-HCC cohort. Figure S4. Kaplan-Meier survival plots based on mutation loads measured by numbers of SNVs, GOHs or LOHs. Figure S5. Analysis based on the recurrent CNVs in 350-kb windows in the 85-HCC cohort. [file 12885_2020_6546_MOESM2_ESM.docx]

**Supplementary Figures**


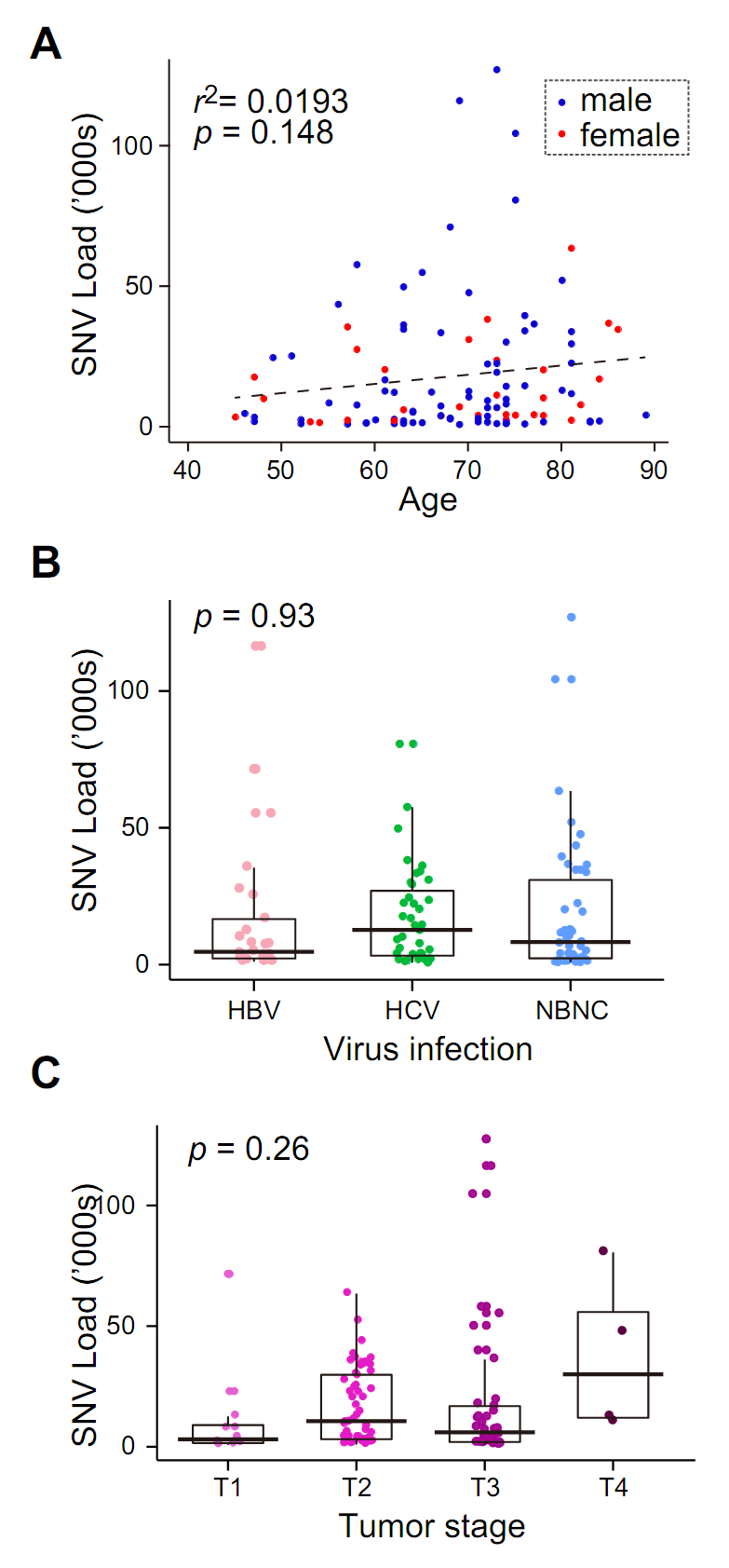


**Figure S1**. Correlation of SNV load with (**a**) age, (**b**) viral status and (**c**) tumor grade in the 110-Liver cohort. The dashed line in (**a**) is the putative result of linear regression (*r^2^* = 0.0193), and gender is shown as male (blue) or female (red). Viral status in (**b**) is shown as HBV (pink), HCV (green) or non-viral (NBNC) (light blue). T1 – T4 in (**c**) refer to tumor grades in the TNM classification system of Union for International Cancer Control.

**Figure S2**. Numbers of patients with different percentages of copy neutral LOHs in the 110-Liver cohort. The total number of copy neutral LOHs detected in each patient sample equaled 100%.


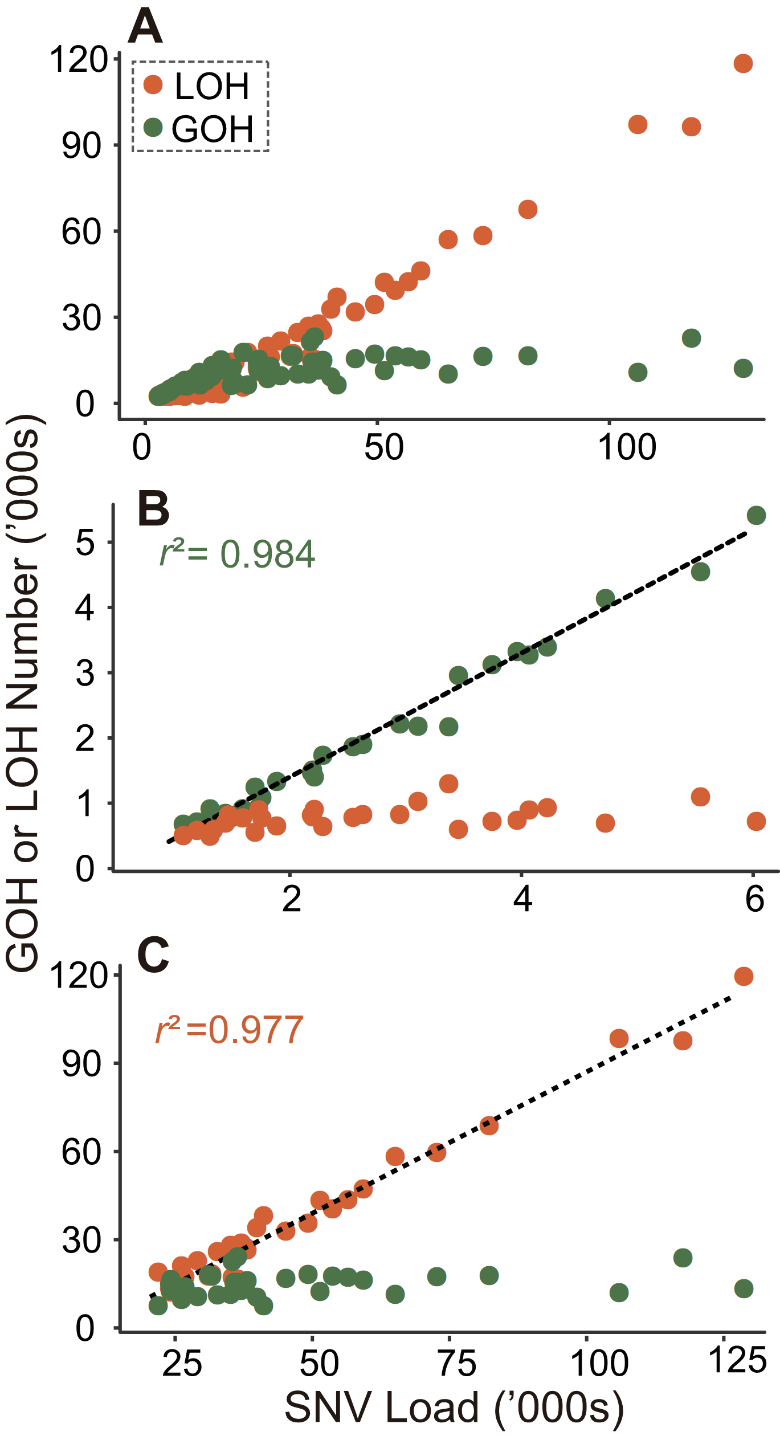


**Figure S3**. Numbers of GOHs and LOHs identified from mapped WGS data for the 85-HCC cohort. GOH (green) and LOH (orange) numbers are shown for (**a**) All 85 samples; (**b**) 28 samples with total SNVs less than 6,000; and (**c**) 36 samples with total SNVs more than 20,000. Linear regression lines with respective coefficient of determination (*r^2^*) calculated from Pearson’s statistic are shown for GOH in (**b**) and for LOH in (**c**).


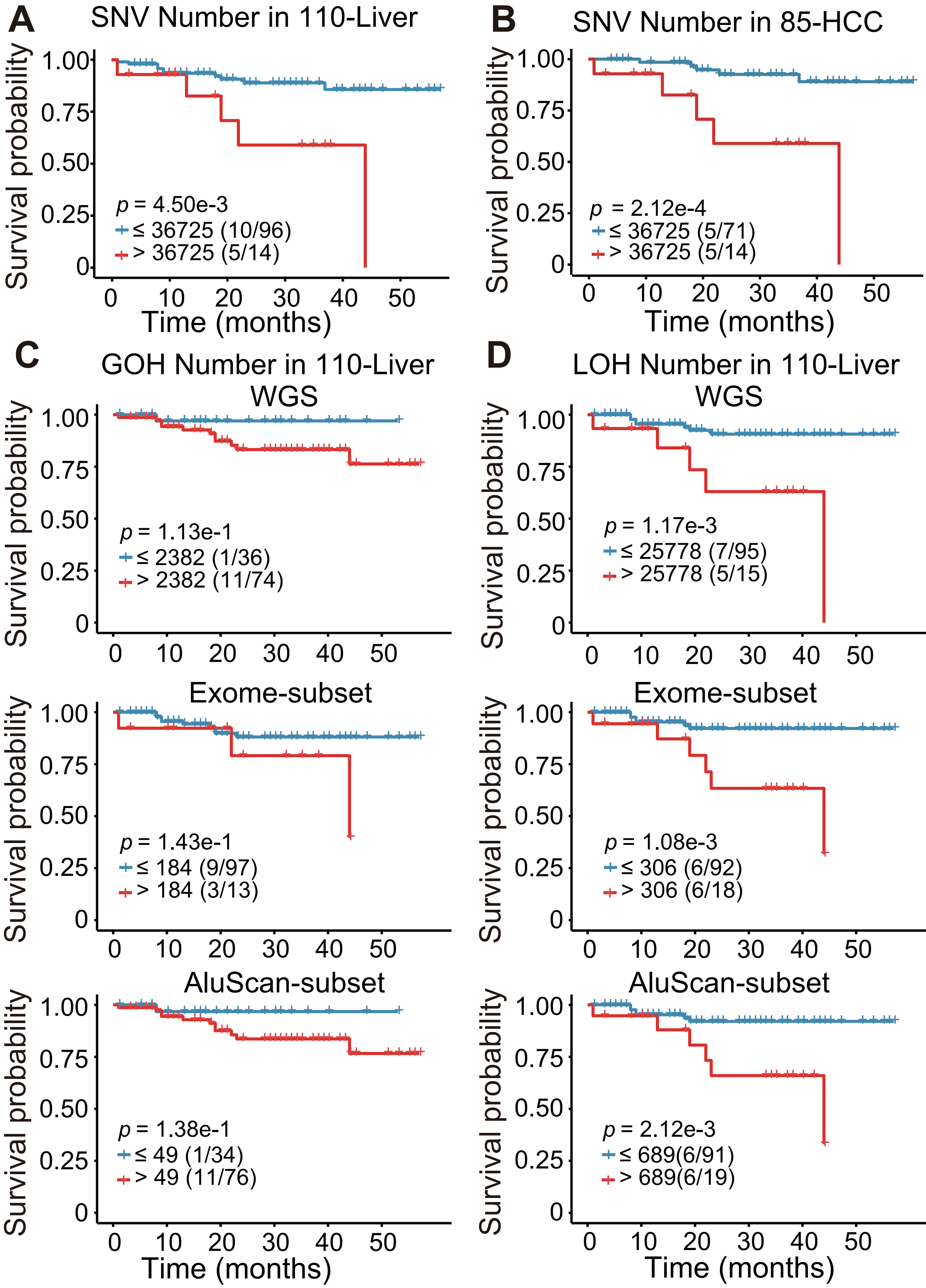


**Figure S4**. Kaplan-Meier survival plots based on mutation loads measured by numbers of SNVs (**A**, **B**), GOHs (**C**) or LOHs (**D**). Survival plots were generated for two subgroups formed by SNV load-based stratification of (**A**) 110 liver cancer patients with fifteen deaths; and (**B**) 85 HCC patients with ten deaths. The cutpoint was defined by the surv-cutpoint function of the survminer R-package. Kaplan-Meier survival plots were generated for two survival-probability subgroups in 110 liver cancer patients stratified based on the numbers of GOH (**C**) and LOH (**D**) in their WGSs (upper panels), Exome-subset (middle panels), and AluScan-subset (lower panels). The total number of patients in each group was shown in the figure. Censored patients were indicated by the tic marks on the survival curves. The optimal cut-points of GOH and LOH numbers employed to divide the patients into two subgroups were identified as the cut-point yielding the lowest p-value in the log-rank test (see ‘*Patient stratification for survival analysis*’ in Methods).


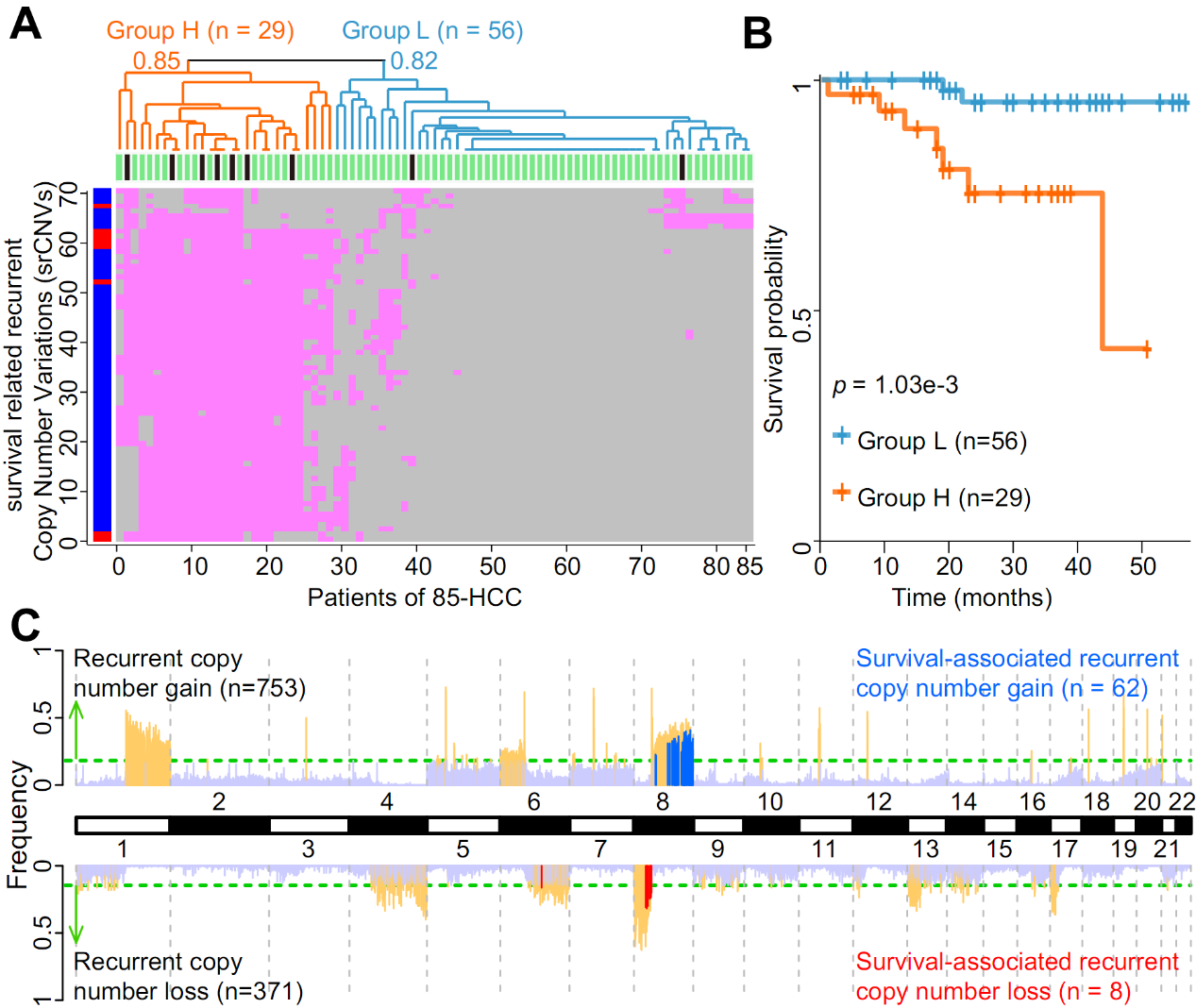


**Figure S5**. Analysis based on the recurrent CNVs in 350-kb windows in the 85-HCC cohort. (**a**) Hierarchical clustering of the 85-HCC cohort based on srCNVs. The approximately unbiased *p*-values after 1,000 times bootstrapping for the high-srCNV group (Group H) and low-srCNV group (Group L) are indicated at the respective nodes. (**b**) Kaplan-Meier survival plots for the two stratified groups. Group H (orange) showed significantly poorer prognosis than Group L (blue) with *p* = 1.03e-3. (**C**) Chromosomal distribution of recurrent CNVs and srCNVs. The frequencies of recurrent CN-gains and CN-losses in 350-kb windows are shown by yellow upward bars and yellow downward bars respectively. The green horizontal lines mark the frequency thresholds for significant recurrence, *p* < 0.05 (0.224 for CN-gains and 0.176 for CN-losses), and the light grey upward and downward bars represent CN-gains and CN-losses respectively that fell below these thresholds. All the srCN-gains (blue bars) and srCN-losses (red bars) were located on chromosomes 6 and 8.
